# Lifestyle and hormonal factors affecting semen quality and sperm DNA integrity: A cross-sectional study

**DOI:** 10.18632/oncoscience.627

**Published:** 2025-09-30

**Authors:** Saniya Imtiyaz Chamanmalik, Rajendra B. Nerli, Pankaja Umarane

**Affiliations:** ^1^Department of Urology, J.N. Medical College, KLE Academy of Higher Education and Research, Belagavi 590010, Karnataka, India; ^2^D.Y. Patil Medical College, Hospital and Research Centre, Kolhapur 416006, Maharashtra, India

**Keywords:** male infertility, lifestyle risk factors, sperm chromatin dispersion test, sperm DNA fragmentation, reproductive hormones

## Abstract

Introduction: Male infertility is a growing public health concern, influenced by modifiable lifestyle factors and hormonal imbalances. However, limited data from India have evaluated their combined impact on semen quality and sperm DNA fragmentation (SDF).

Methods: This cross-sectional study included 278 men aged 21–50 years from a tertiary care centre. Semen analysis was performed according to the WHO’s 6th edition guidelines. The SDF was evaluated using the Sperm Chromatin Dispersion (SCD) test. Lifestyle details, occupational exposures, and hormonal profiles (FSH, LH, testosterone, AMH, prolactin) were gathered through structured interviews and laboratory testing. Statistical analysis was conducted using SPSS, with *p* < 0.05 regarded as statistically significant.

Results: Men aged >40 years showed a significantly elevated SDF (*p* = 0.038), although no significant differences were observed in conventional semen parameters. Tobacco and alcohol use were strongly associated with reduced sperm concentration, motility, and morphology (*p* < 0.001). Alcohol use was also associated with an increased SDF (*p* = 0.023). Abnormal BMI was correlated with poorer semen quality and higher SDF (*p* < 0.001). Occupational heat exposure significantly contributed to elevated SDF levels (*p* = 0.013). Hormonal analysis showed that low testosterone and elevated prolactin levels were associated with abnormal semen profiles, whereas low AMH levels were significantly correlated with increased SDF (*p* = 0.011).

Conclusions: Lifestyle habits and hormonal imbalances significantly affect the fertility of men. Clinical evaluation should be integrated into infertility assessments and counselling.

## INTRODUCTION

Infertility is a major global health concern, affecting an estimated 15–20% of couples worldwide, with male factors contributing to nearly half of these cases [1, 2]. According to the World Health Organization (WHO), approximately one in six individuals will experience infertility at some point in their lives, underscoring the increasing burden on reproductive health services worldwide [3]. In India, the prevalence of infertility among married couples is estimated to range between 10% and 15%, a figure that continues to rise due to factors such as urbanization, delayed parenthood, and increasingly sedentary lifestyles [4, 5].

Over the past few decades, a global decline in semen quality has been observed, which is attributed to the complex interplay between modifiable and non-modifiable factors [6, 7]. Non-modifiable contributors include advancing paternal age, genetic predisposition, and environmental exposure, particularly to endocrine-disrupting chemicals (EDCs) and occupational heat stress. Modifiable lifestyle factors, such as elevated body mass index (BMI), tobacco and alcohol use, poor dietary patterns, physical inactivity, and psychological stress, are strongly linked to hormonal imbalances and oxidative stress, both of which adversely affect spermatogenesis and semen quality [8–10].

Among these factors, obesity has garnered significant attention because of its detrimental effects on male fertility, including hormonal disruption and increased oxidative stress. Excess adipose tissue can elevate scrotal temperature, reduce testosterone levels, and promote the generation of reactive oxygen species (ROS), ultimately impairing sperm concentration, motility, morphology, and DNA integrity [11]. Similarly, chronic tobacco use introduces toxins and ROS that damage sperm membranes and DNA, whereas alcohol disrupts the hypothalamic-pituitary-gonadal (HPG) axis, further impairing spermatogenesis [12, 13].

In addition to lifestyle influences, hormonal abnormalities, such as altered levels of follicle-stimulating hormone (FSH), luteinizing hormone (LH), testosterone, anti-Müllerian hormone (AMH), and prolactin, are frequently observed in men with infertility. These endocrine markers not only reflect testicular function but are also associated with sperm chromatin instability and DNA fragmentation [14, 15].

Sperm DNA fragmentation (SDF) has emerged as a key molecular biomarker for the evaluation of sperm integrity and fertility. Elevated SDF levels have been linked to lower fertilization rates, compromised embryo development, recurrent pregnancy loss, and poor outcomes in assisted reproductive technology (ART) [16, 17].

Despite the growing awareness, few studies in India have comprehensively examined the combined effects of lifestyle habits and hormonal profiles on standard semen parameters and sperm DNA fragmentation. Given the influence of region-specific cultural, dietary, and environmental factors, generating local evidence is essential for guiding clinical decision-making in the management of male infertility.

Therefore, this study aimed to evaluate the associations among modifiable lifestyle factors (BMI, tobacco use, and alcohol use), reproductive hormone levels, semen quality, and sperm DNA integrity in a cohort of infertile Indian men. By addressing this gap, this study seeks to inform early screening strategies and support personalized preventive approaches for male reproductive health.

## RESULTS

### Distribution of semen diagnostic review among study participants

Of the 293 male participants, 15 with azoospermia were excluded from the analysis. Among the remaining 278 participants, 145 (52.2%) had normal semen parameters and 133 (47.8%) had altered semen parameters. The data demonstrated that while there was no significant association between male age and conventional semen diagnostic parameters (*p* = 0.911), there was a statistically significant association between age and sperm DNA fragmentation (*p* = 0.038). Men aged 31–40 years formed the largest group with both normal (65.5%) and altered (61.65%) semen profiles, indicating that age alone did not significantly affect the traditional semen quality metrics. However, sperm DNA fragmentation was higher in the older age groups, particularly 41–50 years (18.9%) and 51 years and above (2.7%), indicating that sperm DNA integrity declines with age. Despite younger men (21–30 years) showing lower fragmentation (18.2%), these results suggest that aging may not significantly affect semen volume or motility but rather impair genetic quality, which is crucial for successful conception and healthy embryo development. (Table 1)

**Table 1:** Effects of male age on conventional semen parameters and sperm DNA fragmentation index

Male age	Diagnostic review				Sperm DNA fragmentation			
	Normal		Alter		No		Yes	
	*N*	%	*N*	%	*N*	%	*N*	%
21–30	27	18.6	29	21.8	29	22.3	27	18.2
31–40	95	65.5	82	61.65	88	67.7	89	60.1
41–50	21	14.5	20	15.03	13	10.0	28	18.9
51 and Above	2	1.4	2	1.5	0	0.0	4	2.7
*P*-value	.911a				.038^*^			

Values are presented as the number of individuals (*n*) and percentages in parentheses. *a* Chi-square test; ^*^p < 0.05 considered statistically significant.

**Figure 1:**
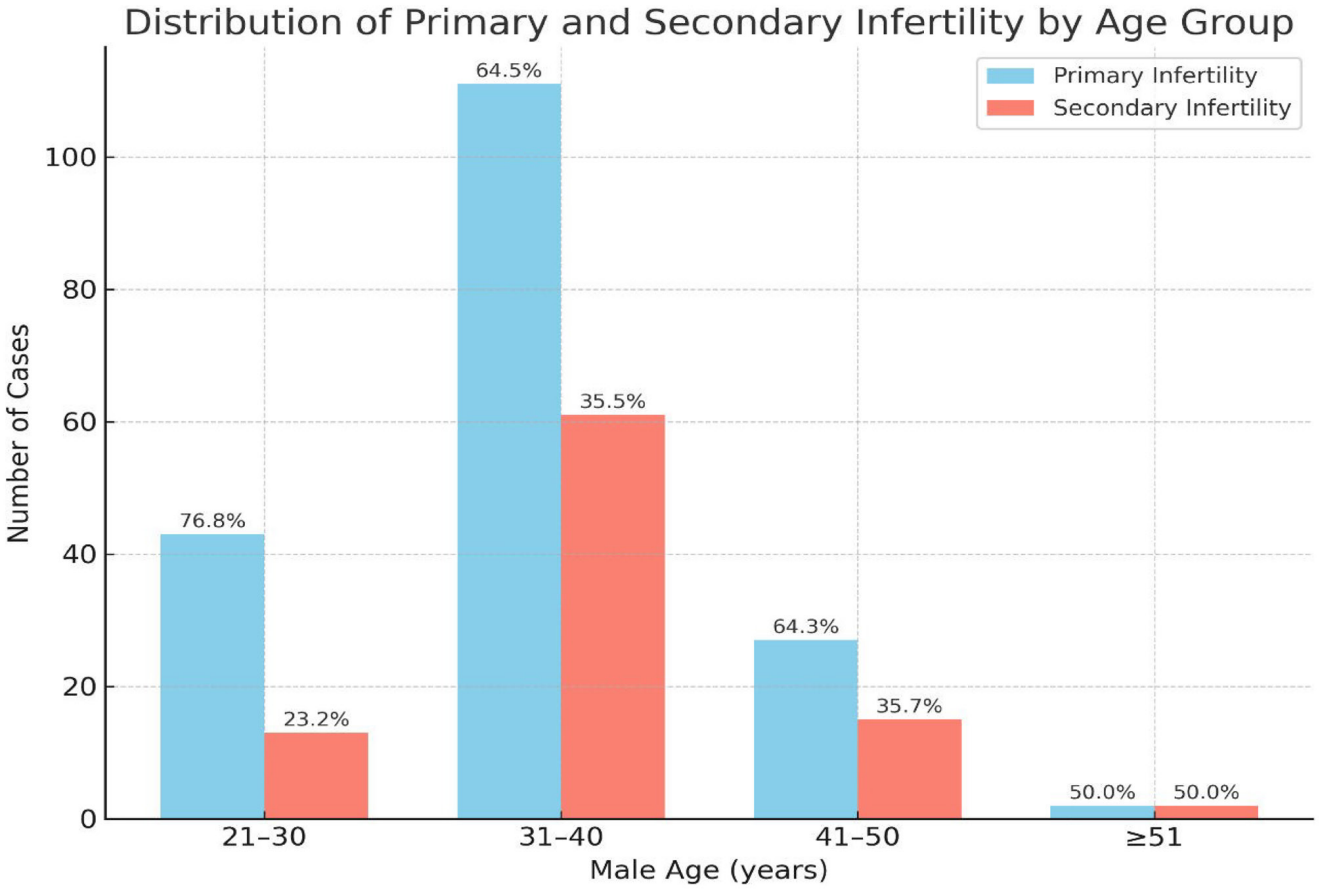
Distribution of primary and secondary infertility by male age group. Primary infertility was more prevalent than secondary infertility in all age categories.

Figure 1 shows the distribution of primary and secondary infertility across different male age groups. The majority of cases of both primary (63.6%) and secondary infertility (63.8%) were observed in the 31–40 years age group. Primary infertility was more prevalent overall (*n* = 173), with most cases occurring in younger age groups, particularly in those aged 21–40 years old. In contrast, secondary infertility (*n* = 105) was more common among men aged 41–50 years (29.5%), indicating a possible age-related effect on fertility after a previous conception.

**Figure 2:**
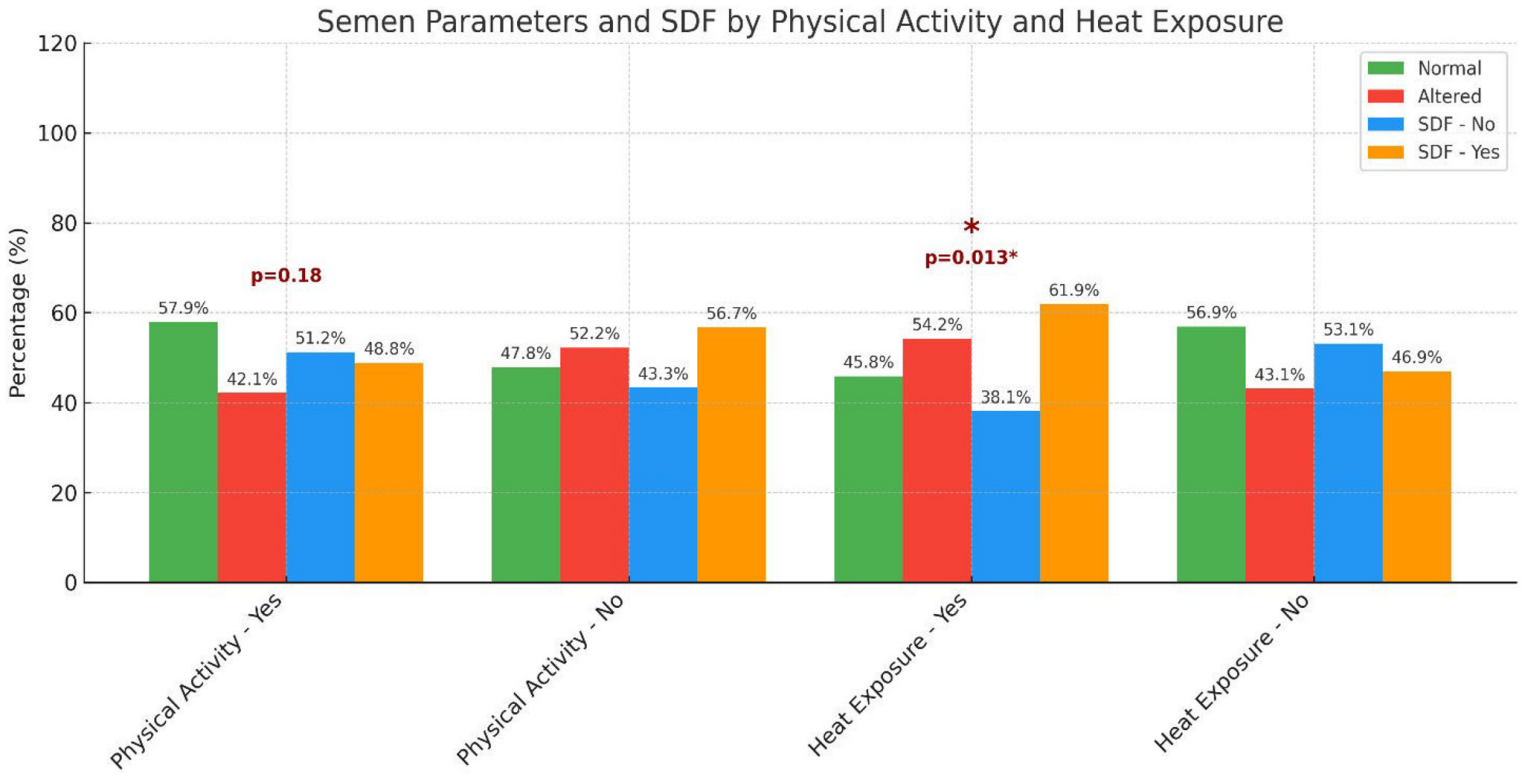
Distribution of semen parameters and sperm DNA fragmentation (SDF) according to physical activity and heat exposure. A significant association was observed between heat exposure and UPE (^*^p = 0.013), whereas physical activity showed no significant effect (*p* = 0.18).

Figure 2 shows the relationship between physical activity, heat exposure, semen parameters, and sperm DNA fragmentation (SDF). No significant association was observed between physical activity and altered semen parameters or SDF (*p* = 0.18); however, occupational heat exposure had a significant effect on SDF (*p* = 0.013). Specifically, 61.9% of men with heat exposure had elevated SDF levels compared to 46.9% of non-exposed men. These findings suggest that environmental heat exposure may negatively affect sperm DNA integrity, potentially affecting male fertility.

Data in Table 2 indicate that tobacco consumption is significantly associated with impaired semen quality. Notably, individuals in the altered semen parameter group showed a statistically significant marked reduction in sperm concentration, total sperm number, motility, and normal morphology. These findings highlight the detrimental effects of tobacco use on key aspects of spermatogenesis and sperm function. Sperm DNA fragmentation (SDF) was also significantly higher in the altered group, suggesting that tobacco may contribute to increased sperm DNA damage, which is critical given its association with reduced fertility, poor embryo development, and compromised assisted reproduction outcomes. Although midpiece defects approached statistical significance, indicating a possible trend toward structural sperm damage, parameters such as semen volume, head and tail defects, and progressive motility did not show significant differences. This suggests that although tobacco affects several important semen parameters, its impact may vary across different aspects of sperm health.

**Table 2:** Comparison of semen parameters between normal and altered groups with tobacco consumption

Semen parameter (Tobacco consumption)	Normal (*N* = 42) (Mean ± SD)	Altered (*N* = 58) (Mean ± SD)	*P*-value
**Volume (mL)**	2.4 ± 0.94	2.5 ± 1.09	0.399
**Concentration (M/mL)**	98.47 ± 49.71	31.94 ± 41.94	<0.001^*^
**Sperm number**	201.1 ± 151.7	57.97 ± 84.6	<0.001^*^
**Motile**	77.2 ± 12.3	45.03 ± 15.83	<0.001^*^
**Progressive motile**	65.66 ± 67.99	107.00 ± 602.49	0.659
**Total morphology >4%**	6.64 ± 2.78	3.72 ± 3.00	<0.001^*^
**Head defects (%)**	86.05 ± 18.74	80.47 ± 29.22	0.28
**Midpiece defects (%)**	50.45 ± 15.73	43.12 ± 20.33	0.054^*^
**Tail defects (%)**	7.50 ± 16.95	3.67 ± 6.53	0.12
**Sperm DNA fragmentation**	0.25 ± 0.44	0.32 ± 0.47	<0.438

Values are presented as mean ± standard deviation, and *p*-values were calculated using independent sample tests. Statistical significance was set at ^*^P < 0.05.

Table 3 presents a comparative analysis of semen quality in males with a history of alcohol consumption, categorized into normal (*N* = 16) and altered (*N* = 14) semen profile groups. Significant reductions in sperm concentration (*p* < 0.001), total sperm count (*p* < 0.007), motility (*p* < 0.001), and progressive motility (*p* < 0.007) were observed in the altered group. These findings indicate that alcohol use is linked to marked impairment in both the quantitative and functional aspects of sperm. Sperm DNA fragmentation was also significantly higher in the altered group (*p* < 0.023), suggesting potential genetic instability in sperm cells. No statistically significant differences were observed in morphology, head defects, midpiece, or tail structure, although the values indicated a trend of impairment.

**Table 3:** Comparison of semen parameters between normal and altered groups with alcohol consumption

Semen parameter	Normal (*N* = 16) (Mean ± SD)	Altered (*N* = 14) (Mean ± SD)	*P*-value
**Volume (mL)**	2.31 ± 1.08	2.17 ± 0.77	0.7
**Concentration (M/mL)**	91.06 ± 48.11	28.63 ± 37.43	<0.007^*^
**Sperm number**	179.22 ± 141.39	57.45 ± 74.21	<0.001^*^
**Motile (%)**	75.30 ± 17.02	44.44 ± 23.11	<0.001^*^
**Progressive motile (%)**	49.16 ± 11.95	30.08 ± 22.75	<0.007^*^
**Total morphology >4%**	5.38 ± 1.20	4.61 ± 1.84	0.182
**Head defects (%)**	81.38 ± 31.58	88.14 ± 17.66	0.484
**Midpiece defects (%)**	43.00 ± 19.04	48.50 ± 20.02	0.447
**Tail defects (%)**	5.44 ± 9.31	2.43 ± 2.79	0.255
**Sperm DNA fragmentation**	0.38 ± 0.50	0.79 ± 0.43	0.023^*^

Values are presented as mean ± standard deviation unless otherwise specified. Statistical significance was set at ^*^P < 0.05.

Comparative analysis of semen parameters between individuals with normal and altered profiles in Table 4, demonstrated significant deterioration in sperm quality among the altered group, which may be closely associated with adverse lifestyle habits such as tobacco use, smoking, and alcohol consumption. Men with altered parameters showed markedly lower sperm concentration, motility, and total sperm count (*p* < 0.001), as well as reduced normal morphology and increased head defects (*p* < 0.001 and *p* < 0.03, respectively). Progressive motility and sperm DNA fragmentation also trended towards significance (*p* < 0.056 and *p* < 0.051, respectively), suggesting underlying cellular and genetic damage. These impairments are consistent with the known detrimental effects of tobacco toxins, oxidative stress from smoking, and alcohol-induced hormonal disruption, all of which compromise spermatogenesis, damage sperm DNA, and alter sperm morphology. Although semen volume, midpiece, and tail defects did not show significant variation, and the overall pattern supports a strong association between multiple unhealthy habits and male reproductive dysfunction. These findings underscore the critical need for lifestyle modifications in individuals facing fertility challenges.

**Table 4:** Comparison of semen parameters in males with multiple lifestyle habits: normal vs. altered semen profiles

Semen parameter	Normal (*N* = 19) (Mean ± SD)	Altered (*N* = 27) (Mean ± SD)	*P*-value
**Volume (mL)**	2.21 ± 0.69	2.32 ± 1.14	0.71
**Concentration (M/mL)**	88.11 ± 41.10	25.15 ± 32.50	<0.001^*^
**Sperm number**	189.85 ± 84.61	58.73 ± 78.42	<0.001^*^
**Motile (%)**	72.76 ± 20.63	34.89 ± 21.40	<0.001^*^
**Progressive motile (%)**	89.44 ± 333.09	95.69 ± 544.29	0.056^*^
**Total morphology >4%**	6.42 ± 2.78	3.40 ± 2.85	<0.001^*^
**Head defects (%)**	90.37 ± 7.82	73.27 ± 34.41	0.03^*^
**Midpiece defects (%)**	46.16 ± 8.14	38.19 ± 20.61	0.119
**Tail defects (%)**	6.53 ± 7.88	4.96 ± 7.05	0.488
**Sperm DNA fragmentation**	0.95 ± 0.23	0.74 ± 0.45	0.051^*^

Values are presented as mean ± standard deviation unless otherwise specified. Statistical significance was set at ^*^P < 0.05.

Analysis of semen parameters in individuals without any reported lifestyle habits (e.g., tobacco use, smoking, or alcohol consumption) revealed significant differences between those with normal and altered semen profiles (Table 5). Despite the absence of lifestyle-related risk factors, the altered group showed significantly lower sperm concentration (38.42 ± 39.74 M/ml vs. 108.62 ± 46.00 M/ml, *p* < 0.001), total sperm number (*p* < 0.001), motility (*p* < 0.001), progressive motility (*p* < 0.008), and normal morphology (*p* = 0.005), which are all critical markers of male fertility. These findings indicate that even in the absence of harmful habits, intrinsic or idiopathic factors may lead to poor semen quality. No significant differences were observed in semen volume, head defects, midpiece defects, tail defects, or DNA fragmentation, suggesting that some structural or genetic aspects of sperm may remain unaffected in non-exposed individuals. These results highlight the multifactorial nature of male infertility and emphasize that poor semen quality can exist independently of external lifestyle factors.

**Table 5:** Comparison of semen parameters between habit-free males

Semen parameter (None)	Normal (*N* = 68) (Mean ± SD)	Altered (*N* = 34) (Mean ± SD)	*P*-value
**Volume (mL)**	2.58 ± 2.88	2.03 ± 0.87	0.28
**Concentration (M/mL)**	108.62 ± 46.00	38.42 ± 39.74	<0.001^*^
**Sperm number**	238.32 ± 127.72	77.57 ± 74.87	<0.001^*^
**Motile (%)**	79.94 ± 13.34	48.99 ± 18.75	<0.001^*^
**Progressive motile (%)**	49.91 ± 14.48	37.14 ± 33.25	<0.008^*^
**Total morphology >4%**	6.31 ± 2.49	4.74 ± 2.83	<0.005^*^
**Head defects (%)**	84.55 ± 22.24	86.53 ± 20.30	0.665
**Midpiece defects (%)**	48.63 ± 15.81	47.41 ± 19.45	0.737
**Tail defects (%)**	3.84 ± 6.08	7.50 ± 17.64	0.128
**Sperm DNA fragmentation**	0.571 ± 0.501	0.707 ± 0.459	0.164

Values are presented as mean ± standard deviation unless otherwise specified. Statistical significance was set at ^*^P < 0.05.

Odds ratio (OR) analysis highlighted the significant impact of various lifestyle habits on the male reproductive health (Table 6). Individuals who engaged in multiple habits (such as smoking, alcohol use, or tobacco chewing) were 2.84 times more likely to exhibit altered semen parameters or elevated sperm DNA fragmentation than compared to those with no such habits compared to those with no such habits (*p* < 0.004). Smoking alone was associated with a 2.36-fold increased risk (*p* < 0.007), whereas tobacco chewing showed the strongest association, with users being 4.22 times more likely to have impaired semen quality (*p* < 0.002). Although alcohol use showed an odds ratio of 1.75, the association was not statistically significant (*p* = 0.185), suggesting that alcohol use alone may not independently predict altered reproductive outcomes in this cohort. The 95% confidence intervals for smoking, tobacco use, and multiple habits did not cross one, reinforcing the reliability of these associations. Overall, the data strongly suggest that modifiable lifestyle factors, particularly smoking and tobacco chewing, significantly contribute to the risk of male infertility.

**Table 6:** Odds ratio relating lifestyle habits

Habits	B	*P*-value	OR	95% C.I. for OR	
				Lower	Upper
**None**			1		
**Multiple habits**	1.045	**0.004** ^*^	2.84	1.38	5.82
**Alcohol**	0.56	0.185	1.75	0.76	4.00
**Smoking**	0.86	**0.007** ^*^	2.36	1.27	4.39
**Tobacco chewing**	1.44	**0.002** ^*^	4.22	1.72	10.31

Odds ratios (OR) and 95% confidence intervals (CI) were calculated using logistic regression, with ‘None’ (no habits) as the reference group. Significant associations were observed among smoking, tobacco chewing, and multiple habits. Statistical significance was set at ^*^P < 0.05.

**Figure 3:**
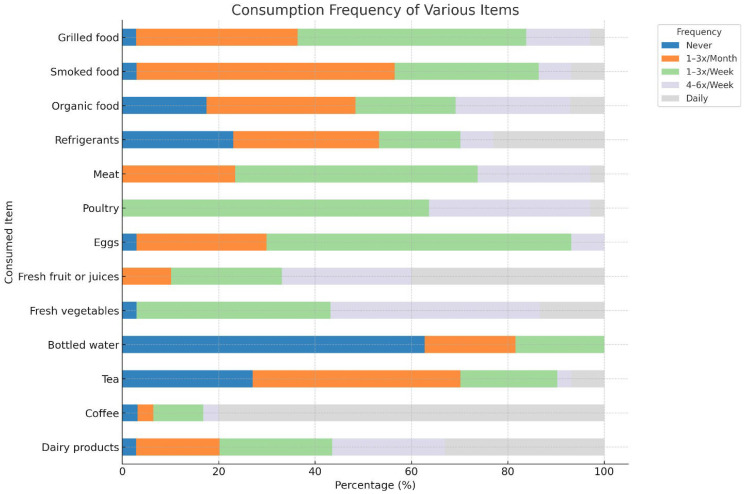
Frequency of consumption of various foods and beverages. Stacked bars represent the percentage of participants reporting each frequency category: never (dark blue), 1–3×/month (orange), 1–3×/week (green), 4–6×/week (purple), and daily (grey).

Figure 3 illustrates dietary patterns among participants with potential implications for fertility: Nearly 80% of the participants consumed tea daily, which provided antioxidants, although excess caffeine can affect semen quality. One-third of the participants consumed dairy products daily, which supports hormonal balance and spermatogenesis. The intake of fresh vegetables and fruits was adequate, with over 40% consuming vegetables 4–6 times a week and nearly 40% consuming fruits daily, offering antioxidants essential for sperm DNA integrity. The use of bottled water was low (more than 60% had never used it), indicating reliance on other sources. Coffee was moderately consumed (mainly 1–3 times per month), limiting caffeine intake. Weekly consumption of meat, poultry, and eggs ensures sufficient protein, although excessive processed meat may reduce fertility. The organic food intake was low, limiting pesticide-free dietary exposure. Smoked and grilled foods were eaten weekly by 30–50%, posing risks due to polycyclic aromatic hydrocarbons linked to oxidative stress and poor semen quality. Overall, the diet included beneficial foods such as fruits, vegetables, and dairy, along with potentially harmful foods such as smoked and grilled items, emphasizing the need for a balanced, antioxidant-rich diet to support male reproductive health.

### BMI (body mass index)

The BMI distribution among 278 male participants indicated that the majority had normal body weight, with 166 individuals (59.71%) falling within the normal BMI range (18.5–24.9). A significant proportion of the 83 participants (29.86%) were classified as overweight (BMI ≥ 25), whereas a smaller group of 27 individuals (9.71%) was underweight (BMI < 18.5). These data highlight that nearly one-third of the participants were above the normal weight range, which may have implications for their reproductive and metabolic health. The predominance of normal BMI provides a balanced baseline for comparing the semen parameters across different weight categories.

Our study demonstrated a significant association between altered BMI and impaired semen quality across multiple parameters (Table 7). Individuals with altered BMI exhibited markedly reduced sperm concentration (70.21 M/mL vs. 95.34 M/mL), total sperm count (150.76 vs. 210.45), motility (61.33% vs. 78.12%), progressive motility (38.64% vs. 52.34%), and normal morphology (4.89% vs. 5.78%), all with statistically significant *p*-values (*p* < 0.001 for concentration, count, motility, and progressive motility; *p* < 0.005 for morphology). These findings highlight the detrimental effects of increased or abnormal BMI on spermatogenesis and sperm function. Although the semen volume was slightly lower in the altered BMI group (2.3 mL vs. 2.45 mL), this difference was not statistically significant (*p* = 0.065). Similarly, no significant differences were observed in the sperm head, midpiece, or tail defects. However, sperm DNA fragmentation was significantly elevated in the altered BMI group (0.49 vs. 0.38; *P* < 0.001), indicating higher oxidative stress and/or apoptosis in this population. These results support the growing body of evidence linking BMI with male fertility potential and suggest that metabolic dysregulation associated with altered BMI may impair both sperm quality and genomic integrity.

**Table 7:** Association of BMI with semen parameters

Semen parameter	Normal (*n* = 145) Mean ± SD	Altered (*n* = 133) Mean ± SD	*P*-value
**Semen volume (mL)**	2.45 ± 0.60	2.30 ± 0.75	0.065
**Sperm concentration (M/mL)**	95.34 ± 40.12	70.21 ± 35.74	<0.001^*^
**Total sperm count (millions)**	210.45 ± 95.60	150.76 ± 90.42	<0.001^*^
**Motile sperm (%)**	78.12 ± 18.44	61.33 ± 20.25	<0.001^*^
**Progressive motility (%)**	52.34 ± 13.32	38.64 ± 15.76	<0.001^*^
**Morphology (% normal forms >4%)**	5.78 ± 1.72	4.89 ± 2.14	0.005^*^
**Head Defects (%)**	82.76 ± 20.65	79.83 ± 21.10	0.313
**Midpiece Defects (%)**	42.80 ± 16.42	45.73 ± 18.04	0.22
**Tail Defects (%)**	5.10 ± 7.91	6.44 ± 8.33	0.239
**Sperm DNA fragmentation index**	0.38 ± 0.21	0.49 ± 0.28	0.001^*^

Values are presented as mean ± standard deviation unless otherwise specified. Statistical significance was set at ^*^P < 0.05.

A detailed analysis of the hormonal profiles of patients with male fertility issues revealed several important relationships between endocrine factors and clinical outcomes (Table 8). The study population demonstrated generally stable gonadotropin levels, with FSH and LH showing normal concentrations in the vast majority of cases (98.5–98.6% and 91.7–95.9%, respectively), indicating preserved hypothalamic-pituitary-gonadal axis function in most patients. However, testosterone levels showed significant variation, with low concentrations (8.3–10.3%) strongly associated with altered diagnostic status (*p* < 0.004) but not with sperm DNA fragmentation (*p* = 0.948). This dissociation suggests that while testosterone deficiency may affect overall fertility potential, it does not appear to directly contribute to sperm chromatin damage.

**Table 8:** Comparative analysis of reproductive hormone levels in men with normal vs. altered semen parameters and DNA fragmentation status

Hormone	Group	Normal (%)	Altered (%)	*P*-value	SDF: No (%)	SDF: Yes (%)	*P*-value
**FSH**	Low	0.7	1.5	**0.511**	0.8	1.4	**0.508**
	Normal	98.6	98.5		98.5	98.6	
	High	0.7	0		0.8	0	
**LH**	Low	4.1	7.5	**0.273**	5.4	6.1	**0.622**
	Normal	95.9	91.7		94.6	93.2	
	High	0	0.8		0	0.7	
**Testosterone**	Low	10.3	8.3	**0.004** ^*^	9.2	9.5	**0.948**
	Normal	89.7	91.7		90.8	90.5	
	High	0	0		0	0	
**Inhibin**	Low	12.4	12.8	**0.553**	14.6	10.8	**0.34**
	Normal	87.6	87.2		85.4	89.2	
	High	0	0		0	0	
**Estradiol**	Low	7.6	3	**0.926**	3.8	6.8	**0.39**
	Normal	84.1	88.7		89.2	83.8	
	High	8.3	8.3		6.9	9.5	
**Prolactin**	Low	0	3.8	**0.033** ^*^	0.8	2.7	**0.192**
	Normal	97.2	91		96.9	91.9	
	High	2.8	5.3		2.3	5.4	
**AMH**	Low	4.1	3.8	**0.872**	0.8	6.8	**0.011** ^*^
	Normal	95.9	96.2		99.2	93.2	
	High	0	0		0	0	
**TSH**	Low	1.4	2.3	**0.495**	0.8	2.7	**0.307**
	Normal	98.6	97		99.2	96.6	
	High	0	0.8		0	0.7	

Distribution of hormonal levels among men with normal versus altered semen parameters with or without sperm DNA fragmentation (SDF). Statistical significance was set at ^*^P < 0.05. Low testosterone and elevated prolactin levels were significantly associated with altered semen profiles, whereas low AMH levels were significantly associated with increased SDF levels.

The most clinically significant findings were related to three key hormones: prolactin, anti-Müllerian hormone (AMH), and testosterone. Hyperprolactinemia (2.8–5.4%) was significantly associated with an altered diagnostic status (*p* < 0.033), likely through its known suppressive effects on the HPG axis. More importantly, anti-Müllerian hormone (AMH) has emerged as a novel biomarker for sperm DNA integrity, with low AMH levels being significantly more prevalent in men with DNA fragmentation (6.8% vs. 1.8%, *p* < 0.011). This finding suggests that Sertoli cell dysfunction, as reflected by AMH production, may play a role in maintaining chromatin stability during spermiogenesis.

The lack of significant associations with other hormones, including inhibin B, estradiol, and TSH, indicates that these factors may have limited diagnostic value in this context. Collectively, these findings suggest that male fertility evaluation should prioritize the assessment of testosterone, prolactin, and AMH levels, as they appear to be the most clinically relevant endocrine markers of both general fertility status and sperm DNA integrity.

### DISCUSSION

This study aimed to comprehensively explore the associations between lifestyle factors, hormonal levels, and male reproductive outcomes, specifically focusing on conventional semen parameters and sperm DNA fragmentation (SDF) in an Indian population sample. Our findings provide valuable insights into the limited data available on this demographic and highlight both modifiable and intrinsic risk factors affecting fertility in men.

A significant association was observed between advanced paternal age and elevated SDF (*p* < 0.038), despite preserved conventional semen parameters. This aligns with the meta-analysis by Szabó et al. (2023) [18], who identified paternal age >50 years as a significant contributor to increased SDF (MD = 12.58%). These findings indicate that while semen concentration, motility, and morphology may remain within reference limits, genomic integrity declines with advancing age, potentially compromising fertilization potential and embryo development.

Environmental and occupational exposure also influenced sperm quality in the present study. Occupational heat exposure was strongly associated with elevated SDF (*p* < 0.013), supporting the evidence that thermal stress induces oxidative damage and chromatin instability in spermatozoa. Although Ebrahimi et al. (2024) [19] did not observe a similar association, the specificity of thermal exposure in our cohort may explain these differences in results.

Tobacco use was associated with significant reductions in sperm concentration, motility, morphology, and total sperm count (*p* < 0.001), consistent with the findings of Henriques et al. (2023) [20], who attributed these effects to tobacco-induced oxidative stress. Although SDF levels were higher in smokers, the association did not reach statistical significance in our cohort (*p* < 0.164), in contrast to Yang et al. (2019) [21], who demonstrated a strong association. Variability in population characteristics, exposure intensity, and SDF assessment methods may account for these discrepancies. Nonetheless, elevated ROS levels due to smoking are biologically plausible for compromising sperm chromatin integrity.

Alcohol consumption had a pronounced negative effect on semen quality, significantly reducing sperm concentration, total count, motility, and progressive motility, while also increasing SDF (*p* < 0.023). These results are consistent with those of Yang et al. (2019), although Szabó et al. (2023) suggested that the effect of alcohol on SDF might be less clinically relevant. These differences may reflect genetic or regional variations in alcohol metabolism. Importantly, men with combined tobacco and alcohol use showed cumulative detrimental effects, particularly on progressive motility (*p* < 0.056) and SDF (*p* < 0.051), supporting the conclusions of Rotimi et al. (2024) [22], who highlighted the synergistic oxidative stress and hormonal imbalance across lifestyle risk factors.

BMI also emerged as a significant modifiable factor, with both underweight and overweight men showing impaired semen parameters and elevated SDF (*P* < 0.001–0.005). This supports the findings of Rotimi et al. (2024) [22], who identified obesity as a cause of impaired sperm function via HPG axis disruption and oxidative stress. Although Szabó et al. (2023) reported weaker associations, our findings emphasize that metabolic imbalance in either direction can impair DNA stability and reproductive potential in male offspring.

Dietary patterns in our cohort varied, with moderate intake of vegetables and fruits but high consumption of caffeine and grilled foods. Although not statistically significant for dietary analysis, this trend may contribute to suboptimal semen parameters. In contrast, Petre et al. (2023) [23] demonstrated improved sperm quality with adherence to the Mediterranean diet, underscoring the role of nutrition in fertility optimization.

A notable strength of this study was the hormonal analysis performed. Low testosterone (*p* < 0.004) and elevated prolactin (*p* < 0.033) levels were significantly associated with impaired semen profiles, whereas low anti-Müllerian hormone (AMH) levels correlated strongly with elevated SDF (*p* < 0.011). This reinforces AMH as a novel biomarker of male infertility, supported by Aydin et al. (2021) [24], Al-Qahtani et al. (2020) [25], and Duvilla et al. (2008) [26]. Given AMH’s role of AMH in Sertoli cell function and testicular integrity, its utility in evaluating sperm chromatin stability deserves further investigation.

Another strength was the application of the WHO 6th edition (2021) semen analysis guidelines, which updated the reference thresholds using global data and highlighted the limitations of single cutoffs (Paffoni et al., 2022). This partly explains the higher rate of abnormal classification in our cohort. Furthermore, SDF, assessed using the sperm chromatin dispersion (SCD) assay, was consistently elevated in men with adverse lifestyle habits and hormonal imbalances. As SDF is strongly linked to ART failure, implantation issues, and miscarriage, it has growing clinical importance as a diagnostic adjunct in idiopathic infertility and ART-refractory cases [2, 17].

The biological mechanisms underlying these associations are multifactorial. Tobacco and alcohol consumption induce oxidative stress, mitochondrial dysfunction, and DNA strand breakage. Heat exposure and BMI dysregulation exacerbate ROS production and impair spermatogenesis. Hormonal imbalances, particularly low testosterone and AMH levels with elevated prolactin levels, indicate disruption of the hypothalamic–pituitary–gonadal (HPG) axis and Sertoli cell dysfunction, which compromise chromatin packaging and DNA stability. These mechanisms provide a biological rationale for the observed correlations.

It is important to emphasize that our findings demonstrate correlations, rather than causation. For example, although smoking and alcohol use are strongly associated with poorer semen profiles and elevated SDF, causality cannot be confirmed without longitudinal or interventional studies. Confounders, such as genetic predisposition, unmeasured environmental exposures, or concurrent subclinical conditions, may also play a role.

Our findings are consistent with those of studies conducted in Europe and Asia. Yang et al. demonstrated that advanced paternal age and lifestyle factors, such as smoking and alcohol consumption, significantly impair semen quality and reproductive outcomes [27]. Henriques et al. reported similar associations, highlighting oxidative stress as a key biological mechanism linking lifestyle habits and sperm DNA damage [28].

In India, studies by Singh et al. [29] and Patel et al. [30] have reported comparable alterations in semen parameters among infertile men, particularly in relation to smoking, alcohol consumption, and BMI. However, these studies did not evaluate sperm DNA fragmentation (SDF) or anti-Müllerian hormone (AMH) levels. Recently, Bhoi et al. [31] provided a broader geographical analysis of semen profiles across Indian infertility clinics, but without molecular or hormonal insights. By incorporating both hormonal profiling and DNA integrity assessment, our study provides a more comprehensive evaluation of male infertility in India.

## MATERIALS AND METHODS

### Study design

**Figure 4:**
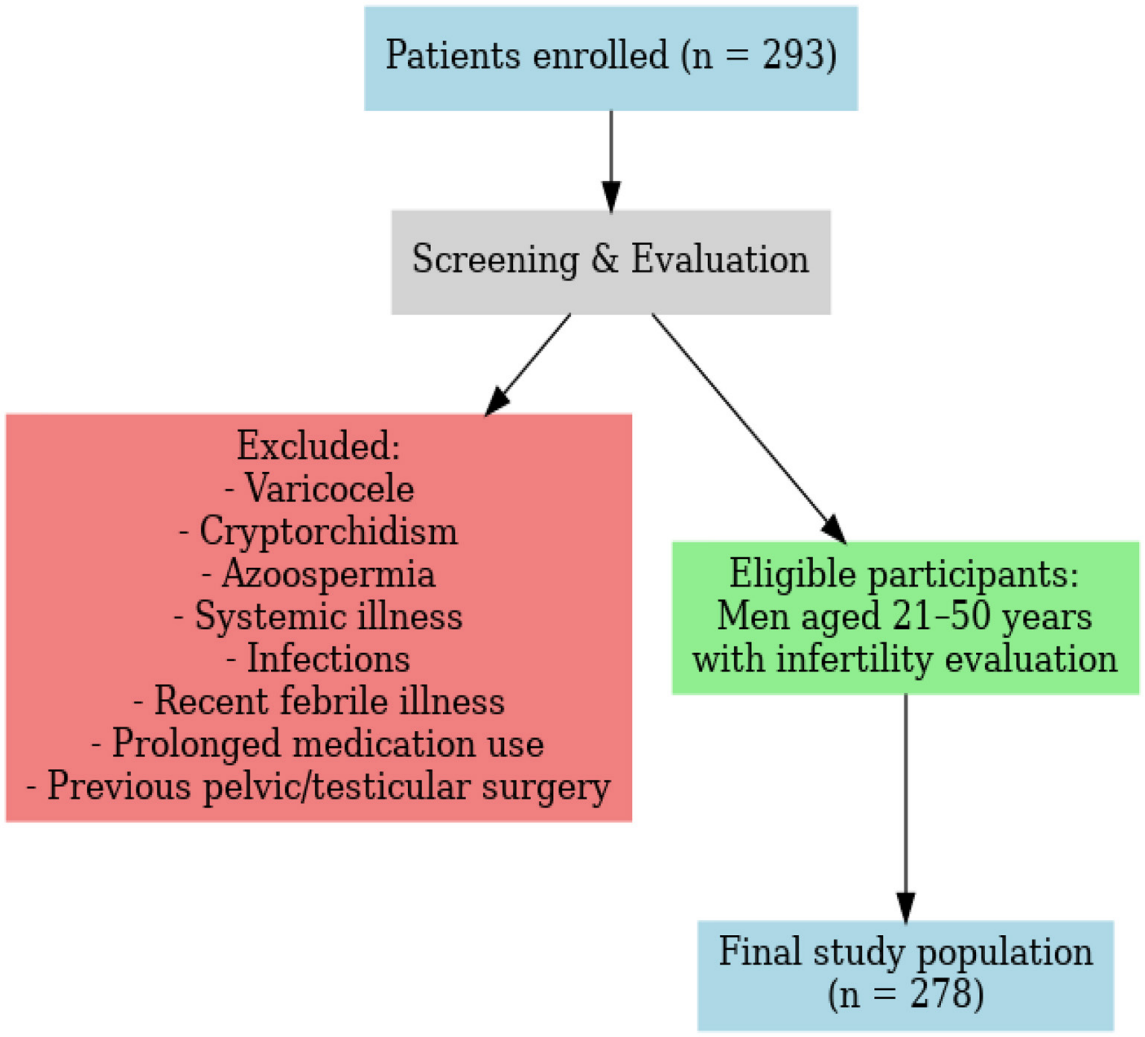
Study population flowchart.

This analytical cross-sectional study was conducted over a period of one year, from March 2023 to March 2024, in the infertility outpatient department of a tertiary care centre. Written informed consent was obtained from all the patients. A total of 293 male patients with infertility-related concerns were initially enrolled. Eligible participants included men aged 21–50 years and above who underwent infertility evaluation, either independently or as part of an infertile couple assessment. To reduce potential confounding factors, individuals with a history of systemic illness, diagnosed infertility-related conditions (varicocele, cryptorchidism, or azoospermia), and those on prolonged medication were excluded from the study. (Figure 4).

### Data collection

After obtaining written informed consent, all participants completed a structured questionnaire to collect detailed sociodemographic and lifestyle data. The questionnaire included items related to age, educational level, occupation, and type of infertility (primary or secondary). It also assessed a range of lifestyle and environmental factors, including tobacco use, alcohol consumption, physical activity, occupational heat exposure, and multiple concurrent habits, as well as participants with no habits. Additionally, participants were asked about their dietary preferences (vegetarian, mixed, or non-vegetarian) and the frequency of consumption of specific food items such as dairy products, eggs, meat, and processed foods. The frequency of food intake was recorded using the following standard scale: never, 1–3 times per month, 1–3 times per week, 4–6 times per week, and daily.

The body mass index (BMI) was calculated using the Quetelet index, which is defined as weight (kg) divided by height (m²) [32]. BMI classification followed the standards established by the Commonwealth Scientific and Industrial Research Organisation (CSIRO) for Caucasian adults aged ≥18 years [33], with the following categories: underweight (<18.5 kg/m²), healthy weight (18.5–24.9 kg/m²), and overweight (≥25.0 kg/m²).

### Hormonal assessment

Fasting venous blood samples (5 mL) were collected from each participant in the early morning (08:00–10:00 h) to minimize diurnal variations. Serum levels of follicle-stimulating hormone (FSH), luteinizing hormone (LH), testosterone, prolactin, and anti-Müllerian hormone (AMH) were quantified following established clinical guidelines [34] to evaluate the endocrine profiles relevant to male fertility. Reference ranges were interpreted according to the WHO (2021) standards and cross-validated with the hospital’s NABL-accredited laboratory values, ensuring both international comparability and local population relevance of the results.

### Semen collection and analysis

**Figure 5:**
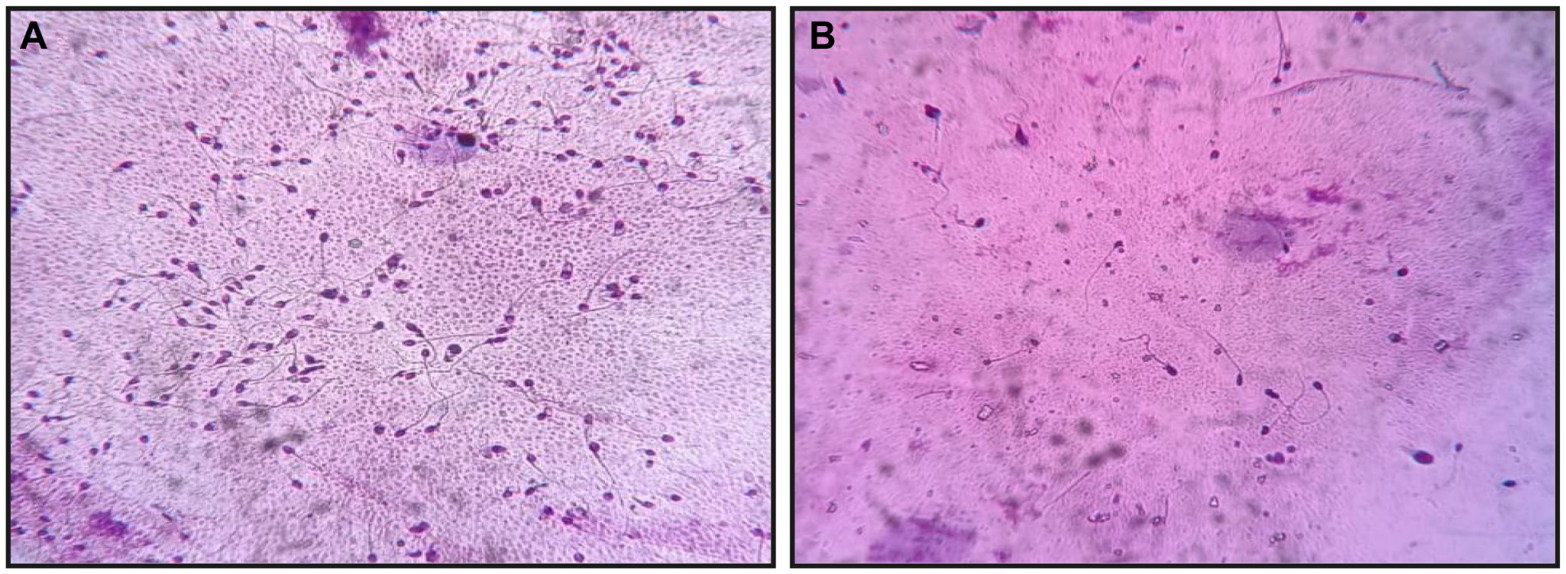
Representative images of semen smears stained with Diff-Quik for sperm morphology assessment at 400× magnification. (**A**) Samples showing normal sperm morphology and higher concentration, with numerous spermatozoa displaying oval heads, intact midpieces, and long, thin tails. (**B**) Samples showing altered sperm morphology and reduced sperm concentration, sparsely distributed spermatozoa with defects such as tapered or amorphous heads, double/coiled tails, detached heads, and midpiece abnormalities.

Semen samples were collected via masturbation after 2–7 days of abstinence and allowed to liquefy at room temperature for 30 min. Semen analysis was performed following the WHO 6th edition (2021) guidelines [35] to evaluate parameters such as volume, pH, concentration, total count, motility, progressive motility, vitality, and morphology of the sperm. Sperm count and motility were assessed using a Makler chamber and confirmed using Computer-Assisted Semen Analysis (CASA) [36]. Based on these findings, the samples were classified into two major groups: normal semen parameters (*n* = 145) and altered semen parameters (*n* = 133) (Figure 5). In total, 278 samples were selected for sperm DNA fragmentation analysis.

### Sperm DNA fragmentation testing

**Figure 6:**
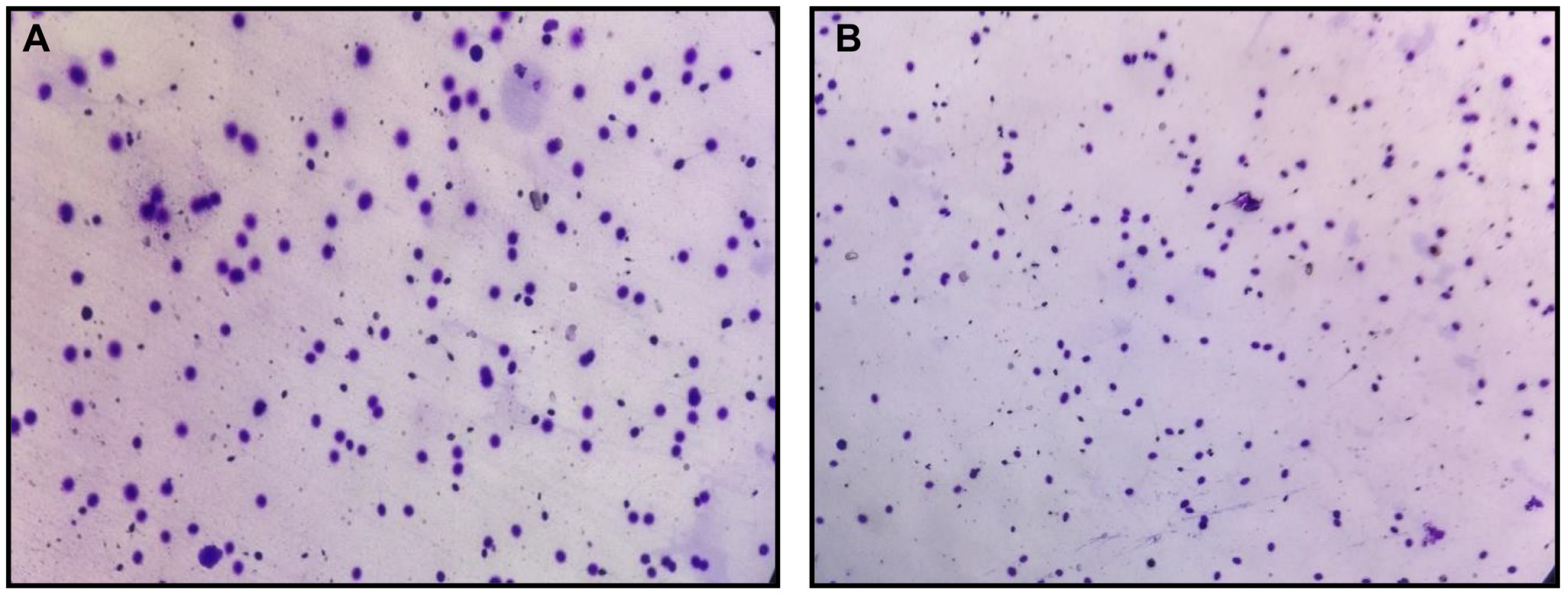
Representative sperm chromatin dispersion (SCD) assay images from infertile male patients were analyzed using a Halosperm^®^ kit and visualized under bright-field microscopy at 400× magnification. (**A**) Samples showing spermatozoa with large or medium halos, indicating intact DNA and low DNA fragmentation. (**B**) Samples with predominantly spermatozoa showing small or no halos, representing high levels of sperm DNA fragmentation.

Sperm DNA fragmentation (SDF) was assessed using the Sperm Chromatin Dispersion (SCD) test, employing a commercially available Halo sperm kit (Cell Life Ref: CL06; Cell Life, Visakhapatnam, India) [37], following the manufacturer’s protocol. Briefly, liquefied semen samples were embedded in agarose microgels on pretreated slides and subjected to acid denaturation, followed by lysis to remove the nuclear proteins. This process allows the relaxation and dispersion of intact DNA loops, whereas fragmented DNA fails to produce halos. The slides were stained and examined under a fluorescence microscope at 400× magnification. For each sample, a minimum of 500 spermatozoa were evaluated per sample. Sperm cells with large, well-formed halos were classified as having intact DNA, whereas those with small, irregular, or no halos were considered to have fragmented DNA (Figure 6) [38].

### Statistical analysis

All statistical analyses were performed using SPSS version 20 (IBM Corp., Armonk, NY, USA). Data are presented as mean ± standard deviation (SD) for continuous variables. Group comparisons were performed using Student’s *t*-test for independent samples. Pearson’s bivariate correlation analysis was used to assess the relationship between sperm DNA fragmentation (SDF) and the continuous semen parameters. Associations between categorical variables, such as smoking and alcohol consumption, were evaluated using Spearman’s rank correlation. The final sample size (*n* = 293) was determined based on the prevalence estimates of male infertility from previous studies and ensured adequate statistical power (>80%) to detect significant associations. A *p*-value of <0.05 was considered statistically significant [39].

## CONCLUSIONS

Considering the comprehensive results and analyses, this study provides strong clinical evidence linking modifiable lifestyle factors, hormonal imbalances, and advanced paternal age with impaired semen quality and elevated sperm DNA fragmentation (SDF). The application of the WHO 6th edition semen analysis criteria enhances the relevance of these findings to current diagnostic standards. Collectively, these results support the need for an integrative approach to male infertility assessment, including routine lifestyle evaluation, hormonal profiling, and SDF testing. Such a multidimensional strategy can improve diagnostic accuracy, inform personalized treatment plans, and enhance the success rates of assisted reproductive technologies (ART).

### Limitations

This study has several limitations. Its cross-sectional design and single-center scope may limit the generalizability of our findings. Additionally, the absence of a fertile control group and lack of longitudinal follow-up data prevented the assessment of pregnancy and ART outcomes.

### Future directions

Future studies should focus on multicenter longitudinal designs involving larger cohorts to confirm these associations and their clinical relevance. Emerging biomarkers, such as AMH, should be explored further. Interventional studies assessing the impact of lifestyle modifications, nutritional interventions, and hormone normalization on semen and DNA quality may provide valuable guidance for improving fertility outcomes.
